# AI in medical education: uses of AI in construction type A MCQs

**DOI:** 10.1186/s12909-024-05250-3

**Published:** 2024-03-06

**Authors:** Assad Ali Rezigalla

**Affiliations:** Department of Anatomy, College of Medicine, University of Bisha, Bisha, 61922 Saudi Arabia

**Keywords:** AI, Construction, MCQs, Item analysis, High quality

## Abstract

**Background:**

The introduction of competency-based education models, student centers, and the increased use of formative assessments have led to demands for high-quality test items to be used in assessments. This study aimed to assess the use of an AI tool to generate MCQs type A and evaluate its quality.

**Methods:**

The study design was cross-sectional analytics conducted from June 2023 to August 2023. This study utilized formative TBL. The AI tool (ChatPdf.com) was selected to generate MCQs type A. The generated items were evaluated using a questionnaire for subject experts and an item (psychometric) analysis. The questionnaire to the subject experts about items was formed based on item quality and rating of item difficulty.

**Results:**

The total number of recurrent staff members as experts was 25, and the questionnaire response rate was 68%. The quality of the items ranged from good to excellent. None of the items had scenarios or vignettes and were direct. According to the expert’s rating, easy items represented 80%, and only two had moderate difficulty (20%). Only one item out of the two moderate difficulties had the same difficulty index. The total number of students participating in TBL was 48. The mean mark was 4.8 ± 1.7 out of 10. The KR20 is 0.68. Most items were of moderately difficult (90%) and only one was difficult (10%). The discrimination index of the items ranged from 0.77 to 0.15. Items with excellent discrimination represented 50% (5), items with good discrimination were 3 (30%), and only one time was poor (10%), and one was none discriminating. The non-functional distractors were 26 (86.7%), and the number of non-functional distractors was four (13.3%). According to distractor analysis, 60% of the items were excellent, and 40% were good. A significant correlation (*p* = 0.4, *r* = 0.30) was found between the difficulty and discrimination indices.

**Conclusion:**

Items constructed using AI had good psychometric properties and quality, measuring higher-order domains. AI allows the construction of many items within a short time. We hope this paper brings the use of AI in item generation and the associated challenges into a multi-layered discussion that will eventually lead to improvements in item generation and assessment in general.

## Background

The introduction of competency-based education models and student centers and the increased use of formative assessment have led to demands for high-quality test items to be used in assessments [1]. Moreover, the popularity of progress and exit tests necessitates using many test items. MCQs are the most commonly used tools in assessment because their reliability and validity are approved, and they can cover a large range of knowledge and knowledge [2–6]. The construction of high-quality MCQs type A has been reported to be difficult and time-consuming [4, 7–9].

Many authors have addressed guidelines for constructing MCQs [2, 9–11]. These guidelines aim to construct high-quality MCQs. In general, these guidelines can be classified as pre- and during construction. Preconstruction guidelines include the presence of a valid blueprint and content material from which the MCQs will be constructed. The most important post-construction (use) guideline, is item analysis because it provides feedback to item constructors about their quality.

The growing need for test items requires new advancements in item construction and generation in addition to the traditional method [1]. Artificial intelligence (AI) can provide such advancements. Artificial intelligence (AI) is commonly applied to computer technologies that mimic or simulate processes supported by human intelligence. Some can perform tasks that involve human interpretation and decision-making [12]. Education is considered the most relevant field of AI application [13]. The use of AI in medical education gained early attention, and the guidelines set by UNESCO aimed to achieve excellence in education [14, 15]. Thus, in education, AI is embedded in many technological innovations that provide learning analytics, recommendations, and diagnosis tools for various ways and purposes [16]. In the field of education, artificial intelligence (AI) isn’t limited to traditional face-to-face teaching and smart learning environments. It is primarily utilized in e-learning to enable automated and personalized learning processes. These processes are based on adaptive learning, machine learning, ontologies, semantic technologies, natural language processing, and deep learning [17]. In medical education, AI applications have been linked to feedback, simulation-based training in medicine, adaptive learning systems, generated assessment tasks, self-assessment, automatic student work scores, and virtual operative assistant creation [18].

It was demonstrated that AI is useful in feedback, assessment, and formative evaluation [17]. AI and AI-driven applications for automated question generation automated questions (AIG)(AQG) are promising advancements. They can significantly simplify the process of generating meaningful and relevant questions from textual educational material. This would facilitate personalized and engaging learning experiences, efficient evaluation of students’ understanding, targeted feedback, and improved educational outcomes for students [19, 20]. In the AIGAQG, content experts are required to articulate the factors that would guide them down a series of different ways to solve a clinical problem [1].

The use of AI applications makes assessments more feasible for maintenance. Jia et al. (2021) proposed a two-step method to improve the quality of automated assessment construction [21]. Step one includes applying a Rough Answer and Key Sentence Tagging scheme. Step two captures the inter-sentence and intra-sentence relations using the answer-guided Graph Convolutional Network for question generation. It has been reported that success in such approaches requires large-scale and relevant datasets for training question-generators [22].

Using AI and AI-driven applications, tools, and techniques can decrease the challenge of traditional methods presented as item construction and item stability [23]. Through AI, it will be easier and more feasible to construct and update items (questions) and form item banks. Different applications and AI tools were used and recommended for item generation. Some of these are costly, need high technical support, and have good results [24–27]. This study used a free and simplified tool to generate items. This study aimed to assess the use of an AI tool to generate MCQs type A and evaluate itstheir quality.

## Methods

The study design was cross-sectional analytic [28] and was conducted at the Department of Basic Medical Sciences (anatomy unit)Department of anatomy from June 2023 to August 2023. The sampling technique is total coverage for the student registered to the musculoskeletal course, College of Medicine (2022–2023). The total number of participants included in the study was 48. The utilized activity was team-based learning (TBL), and the number of items (questions) was 10 according to the regulation and recommendation of TBL conduction.

### The study context

This study utilized formative TBL during the musculoskeletal course (2022–2023). The subject’s TBL content was anatomy, and the title was the posterior abdominal wall. The TBL and topic were chosen to ensure the one-dimensionality of the content material, which can affect internal consistency. TBL is a student-centered instructional methodology for large classes of students divided into small teams of five to seven students [29]. TBL comprises three parts: pre-class, in-class, and post-class [29, 30]. The pre-class part is divided into two teacher and student responsibilities.

Teacher responsibilities include selecting the content material objective, creating the student’s team, providing students with the TBL objectives and the recommended materials or textbooks, and creating the individual readiness test (i-Rat) and group readiness test (g-Rat). The student’s responsibility is to study according to the provided TBL objectives and reading materials. Teams’ creation and assignment are done by the medical education unit in the college, and teams are sustained for an academic year.

The in-class part is divided into two students’ and teachers’ responsibilities. Students’ responsibility is to answer the i-Rat and then, through the g-Rat, with group skills and dynamics, fill the knowledge gaps, if any. The teacher’s responsibilities are to lead the discussion, create new threads, and provide clarification through mini a lecture if needed. The last part of in-class is the application of the learned knowledge. The post-class included appeals or assignments to support knowledge applications. The i-Rat comprises 10 MCQs of type A (reused in the g-Rat). The TBL was conducted by a content expert (atomist) [29–31]. The TBL was managed and processed through institute regulations.

### The selection of an AI tool

The AI tool was selected according to the following criteria:1) available tool, 2) free of charge, 3) easy to use, 4) the tool uses PDF file format (as all the reference books are available as PDF), 5) safe tool, and 6) confidentiality. When applying the criteria, two tools were selected: ChatPdf.com and ChatPDF.ai. ChatPdf.com (https://www.chatpdf.com/) was used for this study.

The ChatPDF.ai uses natural language processing algorithms and deep learning technology to enable users to ‘chat’ with PDF documents. It scans the PDF using optical character recognition and extracts text. The extracted text becomes a data source for the AI to analyze and respond accordingly.

### Generation of question (item)

A PDF file on the posterior abdominal wall was prepared from a standard anatomy book. The PDF file was a section from the anatomy textbook, and it’s one of the recommended books for student teaching and learning.

#### Process of generation

**Fig. 1:**
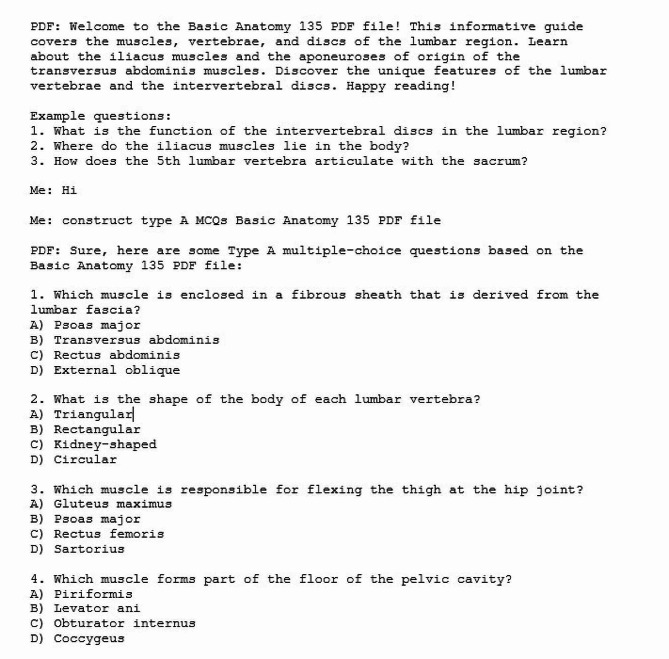
Shows uploading of PDF file and construction of questions

1. The PDF file was uploaded to the tool.
2. The tool used one minute to read and formulate a small PDF file summary.
3. The tool was ordered to construct MCQs type A (Fig. 1).
4. The tool started to construct the questions in order.

### Questions evaluation and assessment

The generated items were evaluated using a questionnaire for subject experts and an item (psychometric) analysis. The questionnaire was tested in a pilot study. The experts included in the pilot study, or the results of the pilot study were not included in the analysis.

The questionnaire to the subject experts about item construction and quality was based on and adapted from the work of Susan and David (2016) and other authors [2, 4, 32] (Table 1).

The rating of item difficulty was assessed based on modified Angoff methods (Table 1) [33, 34].

Inter-rater agreement for the expert rating was limited to 30%. In cases of deviation in ratings, the concerned expert is requested to rerate according to other expert ratings or keep his rates [33].

In the item difficulty part, experts were asked to rate the items as easy, moderately difficult, or difficult using a three-point Likert scale. The questionnaires were distributed to a target group of experts. The target group of experts was selected according to the following criteria: staff members in medical colleges (within the KSA), experience in teaching human anatomy, experience in medical education, and work in a medical college with a similar or equivalent curriculum (problem-based, SPICE). The total number of recurrent staff members was 25.

The generated (item) questions were used in the i-Rat of the TBL. The students’ responses on the i-Rats were verified, marketed, and analyzed, and then an item analysis was generated. Item analysis provides feedback about item construction and its validity and reliability after it appears in an examination [5, 35, 36]. The parameters of item analysis include KR20, difficulty index (p-value), discrimination index, and distractor efficiency (analysis) [5, 8, 37].

Cronbach’s alpha (KR20) was used to estimate exam reliability (internal consistency) [38]. It also describes the dimensionality of the exam and the extent to which the exam measures the same concept or construct [37, 39]. The value of KR20 is affected by the number of items in the exam, the difficulty index, the number of examinees, and performance [37, 39, 40]. A value of 0.7 was reported as acceptable for a short test (less than 50 items) and KR20 of 0.8 for a large exam (more than 50 items test) [41]. It was reported that values > 0.7, 0.6–0.7 is acceptable, 0.5–0.6. poor, < 0.5, and < 0.30, unreliable [42]. The difficulty index (Dif. index) was calculated as the percentage of students who scored the item correctly (absolute difficulty) [37]. The difficulty index ranges from 0 to 100, where higher values indicate easier questions and lower values represent the difficulty of hard items. The range of item difficulty can be categorized as difficult (< 39), moderate (40–80), and easy (> 80) [37, 43]. The item discrimination index (power) (Dis. index) was calculated as the difference between the upper and lower 27% divided by the number of participants in each group. The DIS ranged from 1.0 to − 1.0. A positive DIS indicates that high achievers answered the item more correctly than lower achievers and vice versa [35, 44]. The range of DIS can be categorized into excellent (≥ 0.35), Good (0.25–0.34), Acceptable (0.21–0.24), and poor (≤ 0.20) [45, 46]. For distractor analysis, any distractor selected by less than 5.0% of the students was considered non-functional (NFD), whereas a functional distractor (FD) is one selected by 5% or more [47]. According to the number of NFDs, items were classified as excellent (0NFDs scored 100), Good (1NFDs scored 66.6), moderate (2NFDs scored 33.3), and poor (3NFDs scored 0).

**Table 1:** Shows the checklist for MCQs item quality

NO	Questions	Response
The question as a whole		
1.	Does the question test application of knowledge rather than the recall of isolated facts?	
2.	Does the question satisfy the” cover the options, hand” rule so that an answer can be formulated without seeing the options?	
Options		
3.	Are the options homogeneous in content and phrasing, similar in length, and parallel in structure?	
4.	Does each option follow grammatically and logically from the lead-in?	
5.	Does the correct answer avoid repeating words used in the stem (“clang” clue)?	
6.	Are the distractors phrased to avoid repetition that clues to the correct answer (convergence)?	
7.	Does each option avoid the use of absolute or vague terms, such as” always,” rarely,” usually,” and” never”?	
8.	Has the option set been constructed to avoid using” None of the above” and” All of the above”?	

### Ethical consideration

The study was approved by the Research and Ethics Committee of the College of Medicine, University of Bisha (KSA). The students were informed about the study, and written consent was obtained from the participating students as part of the i-Rat. The students were informed that the TBL was formative and that their participation in the study did not impact their grades or grade point average (GPA)GPA. In addition, the students could attend TBL without including their responses in the study. Permission to use the AI tool was obtained through the support team for the tool.

### Statistical analysis

Students’ responses to the i-Rat and questionnaires were tabulated in MS Excel 2016 and analyzed by SPSS version 25. KR20, difficulty index (absolute difficulty), discrimination index, and distractor efficiency (analysis) were calculated. The relationship between the difficulty index and discrimination index was evaluated by Pearson correlation, and a P-value of < 0.05 was considered statistically significant. The data is presented as a mean ± standard deviation in the form of a table of frequencies.

## Results

The total number of student participants was 48. The mean age and GPA of students were 20.2 ± 0.4 and 3.25 ± 0.35, respectively. All the students were in level three.

### The questionnaire to experts

**Fig. 2:**
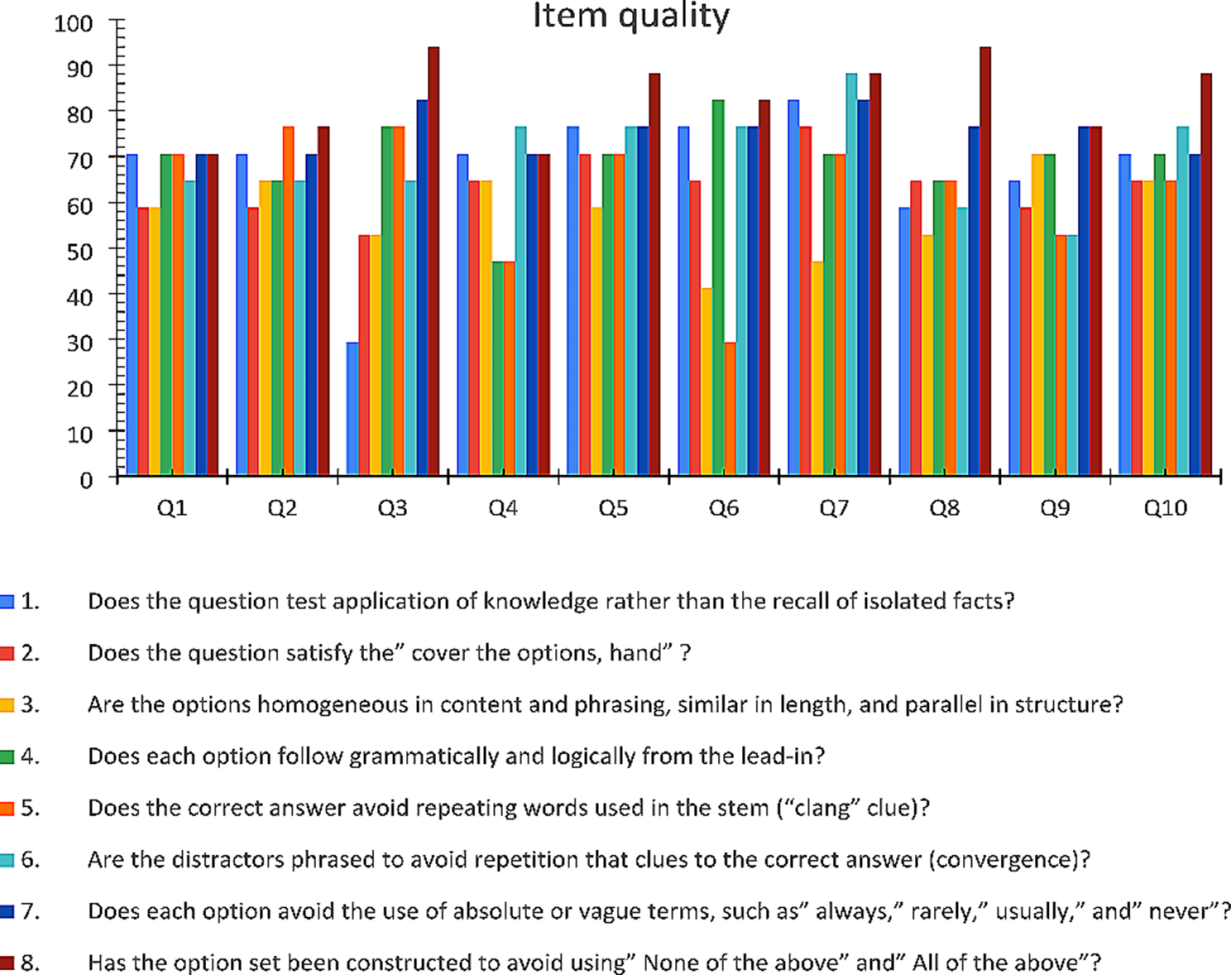
Shows item quality (*n* = 10)

The total number of participating staff members as experts was 25, and the questionnaire response rate was 68%. According to the questionnaire responses, the item quality was good to excellent. None of the items had scenarios or vignettes and were direct. All items tested knowledge and passed the hand cover test. The distractor and correct options followed the grammatical and logical forms. Only one item contained a repetition of words in the stem “clang, clue,” and its difficulty and discrimination indexes were within acceptable levels. The generated items are devoid of absolute words and “none of the above’ (Fig. 2).

According to the expert ratings, the average rating of the items was easy. Easy items represented 80%, and only two had moderate difficulty (20%) (Table 1). Only one item of the two moderate difficulties had the same difficulty index, while the rest had expert ratings that were different from the item difficulty index.

### Item analysis

The total number of students participating in TBL was 48. The mean mark was 4.8 ± 1.7 out of 10. The maximum and minimum marks were 9 and 2, respectively. The KR20 is 0.68. Item analysis of the i-Rat is presented in Table 2. The average difficulty index was 47.7 ± 4.8 (moderate difficulty). Most items were moderately difficult (90%) and only one difficult (10%). The discrimination index of the items ranged from 0.77 to 0.15. The mean discrimination index of i-Rat was 0.41 ± 0.08 (Excellent discrimination). Items with excellent discrimination represented 50% (5), items with good discrimination were 3 (30%), and only one time was poor (10%), and one was none discriminating. The non-discriminating item had a moderately difficult index. The total number of distractors was set to 30. The non-functional distractors were 26 (86.7%), and the number of NFDs was 4 (13.3%). According to distractor analysis, 60% of the items were excellent, and 40% were good (Table 1). Items with deviated parameters in item analysis (one difficult item, one non-discriminating item, and one with poor discrimination power) were checked for possible content, technical, or grammatical flaws.

The Pearson correlation test revealed a significant correlation between the difficulty and discrimination indices (*p* = 0.4, *r* = 0.30).

**Table 2:** Shows item analysis of i-Rat and expert rating of items

Q	A	B	C	D	Dif. index		Dis. index		NFD		Expert rating	
					%	Interp	Score	Interp	NU	Interp	Average	Interp
1	27.1	***56.3***	6.3	8.3	56.3	M.DI	0.31	Good	0	Excellent	1.6	Easy
2	***33.3***	52.1	6.3	6.3	33.3	M.DI	0	None	0	Excellent	1.7	Easy
3	8.3	18.8	***47.9***	25.0	47.9	M.DI	0.77	Excellent	0	Excellent	1.4	Easy
4	25.0	***58.3***	12.5	4.2	58.3	M.DI	0.15	Poor	1	Good	1.5	Easy
5	14.6	***37.5***	4.2	43.8	37.5	M.DI	0.54	Excellent	1	Good	1.8	M.DI
6	***64.6***	14.6	4.2	12.5	64.6	M.DI	0.62	Excellent	1	Good	1.5	Easy
7	***14.6***	66.7	14.6	4.2	14.6	DI	0.31	Good	1	Good	1.5	Easy
8	***58.3***	22.9	6.3	12.5	58.3	M.DI	0.69	Excellent	0	Excellent	1.4	Easy
9	***52.1***	22.9	16.7	8.3	52.1	M.DI	0.38	Excellent	0	Excellent	1.8	M.DI
10	***54.2***	29.2	8.3	8.3	54.2	M.DI	0.30	Good	0	Excellent	1.5	Easy
Average i-Rat					47.7	M.DI	0.41	Excellent	-	-	1.6	Easy

Dif. Index = difficulty index, Dis. Index = Discrimination index, NFD = non-functional distractor, Interp = interpretation, NU = number, M. DI = Moderate difficulty, DI = Difficult

## Discussion

Items in the study generated by the AI tool (ChatPDF.ai) showed good psychometrical analysis. Most items were moderately difficult, and more than half had excellent discrimination and function distractors. The generated items have acceptable average levels of difficulty index and discrimination index. The average rating of items by experts was easy. Despite the expert rating, these findings represented the core objective of the items’ psychometrical analysis for educators. Such findings were reported in anatomy exam [48], community Medicine [49], and multidisciplinary summative exams [50, 51] that were constructed traditionally.

The mean score on the i-Rat was 4.8 ± 1.7. The standard deviation of i-Rat was small. This finding suggests a low degree of variation in the student’s scores on the i-Rat. The students’ scores tended to cluster around the mean, with low dispersion between the minimum and maximum scores. Such clustering of students’ scores may indicate that students’ performance and competence are equal or that the items evaluate students’ knowledge well.

Despite the few items (10), KR20 is 0.68, which is acceptable for in-class assessment [52]. The KR20 is reported to be affected by many factors such as exam time, the number and inter-relation of the items (dimensionality), item difficulty, variations in examinee responses, and the number of examinees [37]. The test time and number of items in the current study were in accordance with recommendations. The TBL content was a single anatomical topic and was conducted according to the standards of TBL conduction [29]. Most of the items were moderately difficult. The total number of participants was 48. As mentioned above, the low level of KR20 was considered to be due to the small number of items. This consideration was supported by the early work of Kehoe (1994). He reported that KR20 values as low as 0.5 are satisfactory for exams formed of 10–15 items [53].

Items with deviated parameters of item analysis were checked, and none contained any content, technical, or grammatical flaws. The mean difficulty index of the i-Rat was 47.7 (moderate difficulty), which is less than that obtained by Escudero et al., Rao et al., and Licona-Chávez et al. for a balanced exam of high-stake [5, 54, 55]. The recommended percentage of items for an ideal difficulty-balanced exam is 5% for easy items, 5% for difficult items, 20% for moderately easy, 20% for moderately difficult, and 50% for average ones [5]. In the current study, the percentage of the moderately difficult items was 90%; meanwhile, the recommended percentage for the balanced exam was 20%.

The mean discrimination index of i-Rat was 0.41, which was higher than that described by Licona-Chávez et al. [5]. Despite one item that is not discriminating, the test has excellent quality to discriminate between higher- and lower-achieving students.

The existing findings indicate a significant positive correlation between difficulty and discrimination indices (*p* = 0.4, *r* = 0.30). Other authors have reported a significant linear and dome-shaped relationship between difficulty and the discrimination index [6, 56]. These reports indicate that an increase in DI is associated with an increase in the discrimination index. Controversially to the current findings, Alareifi (2023) described a strong negative correlation between difficulty and discrimination indices (*p* = 0.00, *r*= -0.936) [57]. Difficult items with no flaws (content, technical, or grammatical flaws) were answered by good (high achievers) rather than low students (high achievers). Logically, difficult items can discriminate between students and have a high discrimination index. A negative correlation between difficulty and discrimination indices indicates an association between easy items and a high discrimination index. Meanwhile, the easy items answered by most examinees will not discriminate between them.

In the current study, experts rated the items as easy, and only two were moderately difficult. The difficulty index of the items was moderate, and only one item was difficult. According to the difficulty index, experts underestimated the item difficulty. There are variations and contradictions in experts’ judgments regarding item difficulty in the literature [58–60]. Experts estimate item difficulty better than the absolute difficulty of items [58]. Additionally, they reported and underestimated the percentage of ease. These discrepancies between the absolute difficulty index and rating could be due to differences in experts’ opinions about items, experiences, and the process of estimating the difficulty of test items [59].

Items generated through AIGAQG have psychometric properties similar to traditionally constructed [1, 61]. Currently, the items generated by AI have acceptable levels of difficulty index, discrimination, and distractor analysis. The KR20 was affected by a small number of items. According to the present findings, items generated with AI in less time need no review regarding language editing, construction, or cost. These findings represent the core objective of decreasing burdens on teachers and providing the requested number of good-quality items. Teachers or human interference will guide data entry by selecting the content materials according to the desired assessment and guaranteeing the quality of items by avoiding overlapping and related technical flaws such as hanging and the presence of technical claws. Teachers will still lead the distribution of items and their ability to assess or judge students.

## Conclusion

Items constructed using AI had good psychometric properties and quality, measuring higher-order domains. AI allows the construction of many items within a short time. We hope this paper brings the use of AI in item generation and the associated challenges into a multi-layered discussion that will eventually lead to improvements in item generation and assessment in general.

### The study limitations

The sample size was small (48 participants). A small number of items. The study context was TBL. We did not test whether the AI tool would generate the same questions (items) on other occasions.

### Recommendation

Conduction of studies on a large group of students with many items. Conduction of a follow-up study to assess the psychometric stability of the generated items. Future research should use AI tools to generate items, including case scenarios and clinical reasoning. This study can form a base for other studies targeting different AI tools and applications to generate or construct different types of items and their psychometric analysis and properties.

All relevant data are within the paper and its Supporting Information files.

The authors have declared that no competing interests exist.

The funders had no role in study design, data collection and analysis, publication decisions, or manuscript preparation.

## Data Availability

The supporting data will be available upon request to the corresponding author.

## Abbreviations

AQG: Automated Question Generation
GPA: Grade Point Average
AI: Artificial Intelligence
MCQ: Multiple Choice Questions
TBL: Team-Based Learning
i-Rat: Individual Readiness Test
g-Rat: Group readiness test Group Readiness Test
KR20: Kuder-Richardson 20
DIF: Difficulty Index
DIS: Discrimination Index
DE: Distractor Efficiency
FD: Functional Distractor
NFD: Non-Functional Distractor
