# Correction: AI in medical education: uses of AI in construction type A MCQs

**DOI:** 10.1186/s12909-024-05297-2

**Published:** 2024-03-21

**Authors:** Assad Ali Rezigalla

**Affiliations:** Department of Anatomy, College of Medicine, University of Bisha, 61922 Bisha , Saudi Arabia

**Correction: Rezigalla BMC Medical Education (2024) 24:247.**

10.1186/s12909-024-05250-3

In the PDF version of the original article [1], the author’s affiliation was missing. The affiliation of the author is Department of Anatomy, College of Medicine, University of Bisha, Bisha 61,922, Saudi Arabia, as shown in this Correction article.

The original article [1] has been corrected.

## References

[1] Rezigalla AA (2024). AI in medical education: uses of AI in construction type A MCQs. BMC Med Educ.

